# Falso valor de HbA_1c_ debido a la variante inusual hemoglobina Petie Salpetriere coheredada con talasemia alfa

**DOI:** 10.1515/almed-2024-0143

**Published:** 2024-11-08

**Authors:** Esperanza Lepe Balsalobre, Gema María Varo Sánchez, Marta Rico Rodríguez, Sandra Fuentes Cantero

**Affiliations:** Servicio de Laboratorio Área de Gestión Sanitaria Norte de Huelva, Hospital de Riotinto, Minas de Riotinto, Huelva, España

**Keywords:** variante, hemoglobina, interferencia, glucosa, diabetes

## Abstract

**Objetivos:**

Describir la variante de hemoglobina que interfiere en el análisis de HbA_1c_ mediante HPLC de intercambio catiónico.

**Caso clínico:**

Paciente de 78 años que acude a consulta de Medicina Interna para una revisión rutinaria, donde se incluye la determinación de HbA_1c_ para la detección de diabetes. El paciente presenta como antecedentes hipertensión y dislipidemia sin presentar síntomas previos sugestivos de diabetes como hiperglucemia, pérdida de peso, polidipsia, poliuria o cansancio. La prueba de detección de la diabetes se realizó mediante determinación de la HbA_1c_ con una columna de HPLC de intercambio catiónico y un analizador de inmunoensayo en el lugar de la asistencia (*point of care*). En la prueba rutinaria de detección de hemoglobinopatías se incluyó un hemograma completo, así como la determinación de HbF y HbA_2_ mediante HPLC de intercambio catiónico y electroforesis capilar (EC). La caracterización de variantes se realizó mediante secuenciación de ADN. Los resultados de HbA_1c_ obtenidos fueron discordantes, la técnica HPLC de intercambio catiónico mostró una elevación de HbA_1c_ de 52 mmol/mol, y el inmunoensayo mostró un nivel normal de 34 mmol/mol. La forma anormal del pico de HbA_1c_ en el cromatograma motivó la realización de la prueba de detección de hemoglobinopatías para investigar la posible interferencia de variantes. Así, se realizó un análisis del gen de la globina, cuyos resultados revelaron la presencia de una variante de hemoglobina llamada “Hb Petie Salpetriere”. Esta variante surge de una sustitución Val→Phe resultante de una mutación de c.103G>T del gen de la beta-globina [BETA34 (B16) Val>Phe; HBB:c.103G>T]

**Conclusiones:**

Se trata del primer caso de detección de la variante Hb Petie Salpetriere en un paciente español. Los resultados obtenidos indican que la variante Hb Petie Salpetriere puede interferir en los resultados de los análisis de HbA_1c_ realizados mediante HPLC de intercambio iónico, no ocurriendo lo mismo con el método de inmunoensayo de aglutinación de látex. Únicamente el HPLC de intercambio iónico reveló la presencia de la variante de la Hb en este caso, lo que indica que se puede detectar una posible variante mediante una revisión meticulosa del cromatograma.

## Introducción

La hemoglobina glicada (HbA_1c_) es la magnitud más utilizada para realizar el control glucémico en pacientes diabéticos. También se utiliza para el diagnóstico de la diabetes. La exactitud de algunos métodos se puede ver afectada por la presencia de hemoglobinopatías. El término hemoglobinopatía incluye todos los trastornos genéticos de la hemoglobina (Hb), esto es, las hemoglobinas talasémicas y anormales. Se estima que, en el año 2000, había 171 millones de personas con diabetes mellitus en el mundo, y se prevé que esta cifra alcanzará los 366 millones en el año 2030. Aproximadamente el 7 % de la población mundial son portadores heterozigóticos de trastornos de la hemoglobina. Las variantes más comunes en todo el mundo, por orden descendente son HbS, HbE, HbC y HbD. Algunas variantes de la hemoglobina están asociadas a patologías, aunque la mayoría permanecen clínicamente silentes y suelen ser detectadas de manera accidental, en ocasiones cuando se mide la HbA_1c_. En casos de heterocigosidad, la supervivencia de los eritrocitos suele ser normal, por lo que la determinación de la HbA_1c_ es adecuada para el control glucémico, siempre que la variante no interfiera con el método empleado o con la unión de la glucosa a la hemoglobina [1, 2].

## Caso clínico

Paciente de 78 años que acude a consulta de Medicina Interna para una revisión rutinaria, donde se incluye la determinación de HbA_1c_ para la detección de la diabetes. El paciente presenta antecedentes de hipertensión y dislipidemia, sin presentar síntomas previos sugestivos de diabetes como hiperglucemia, pérdida de peso, polidipsia, poliuria o cansancio.

**Figura 1:**
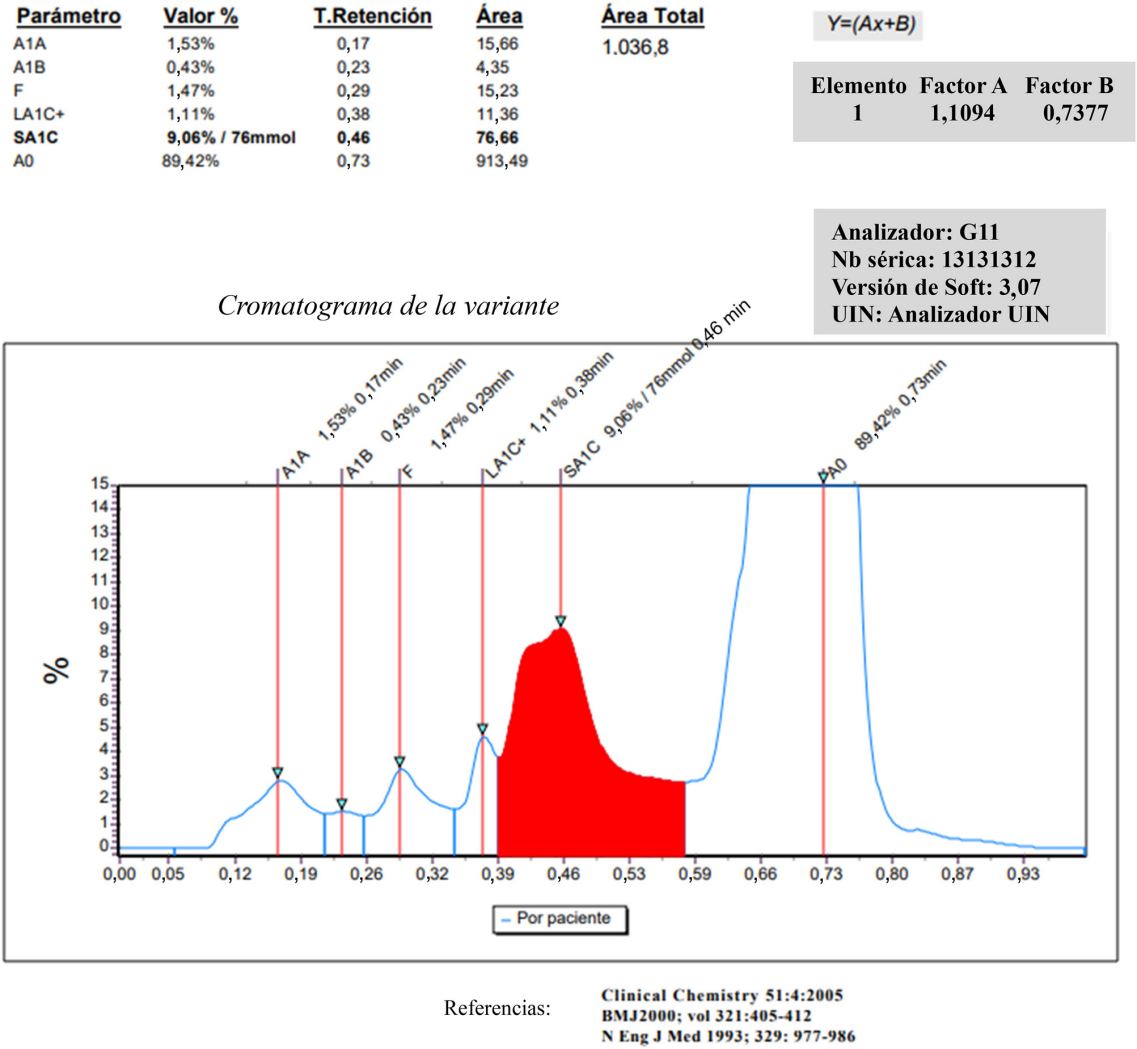
Cromatograma de la variante Hb detectada.

El valor de la HbA_1c_ fue de 77 mmol/mol (9.1 %) obtenido con el analizador de cromatografía líquida de alto rendimiento (HPLC) de intercanbio iónico (Tosoh G11, Horiba, Japan) (Figura 1). Su glucemia en ayunas fue de 5,77 mmol/L (límite superior del intervalo de referencia, 6,1 mmol/L), y la electroforesis de hemoglobina fue normal. Otros resultados obtenidos indicaron: una concentración de hemoglobina de 181 g/L, hematocrito de 0,549 L/L, macrocitosis leve (debido al déficit de vitamina B12 (cobalamina)) (volumen corpuscular medio de 101,7 fL, valores de referencia: 82–96 fL) con hipercromia (hemoglobina corpuscular media de 33,5 pg, valores de referencia: 27–31 pg).

La HbA_1c_ determinada mediante inmunoensayo de aglutinación de látex (Cobas b101, Roche Diagnostics, Suiza) fue de 25 mmol/mol (4,3 %), más acorde a sus valores basales de glucemia en sangre. No se observaron alteraciones significativas en otros parámetros bioquímicos.

Dado que el cromatograma anormal suscitó sospechas de la existencia de una variante de la hemoglobina, las muestras sanguíneas se enviaron para su caracterización molecular a un laboratorio de referencia de talasemia y hemoglobinopatías. Las cadenas de globinas se analizaron mediante HPLC de fase inversa, que reveló la pérdida de un gen alfa en un alelo, debido a la deleción de 3.7 KB (-ALFA 3.7/ALFA ALFA). Además, se realizó un análisis del gen de globina, cuyos resultados revelaron la presencia de una variante de la hemoglobina llamada “Hb Petie Salpetriere”. Esta variante surge de una sustitución Val→Phe debida a una mutación de c.103G>T del gen de la beta-globina [BETA34 (B16) Val>Phe; HBB:c.103G>T] Así mismo, se produjo el descubrimiento casual de una talasemia heterocítica asociada a alfa.

## Discusión

La sospecha de la presencia de una hemoglobinopatía en el paciente tras el análisis de HbA_1c_ condujo a la identificación de una variante estructural de la cadena de beta-globina conocida como ‘Hb Petie Salpetriere’. Tras una revisión de la Base de Datos de Variantes de la Hemoglobina Humana y Talasemia (HbVar), únicamente encontramos dos casos descritos en la literatura: un caso en Japón y otro en Francia. De este modo, nos encontramos ante el primer caso descrito de esta variante en España [3, 4].

La Hb Petie Salpetriere es sustituida en la posición 34 de la cadena beta, que está directamente implicada en el contacto de ɑ1ß1. Una sustitución en esta región provoca un desequilibrio alostérico de la hemoglobina, lo que provoca la pérdida de enlaces de unión en estos contactos, perdiendo la estructura cuaternaria desoxi. El tipo de sustitución de aminoácido, su posición y la proporción de la variante de hemoglobina, influyen en la funcionalidad de la molécula debido a la implicación del residuo 34 en la unión de las cadenas de beta globina con el grupo hemo. Concretamente, la Hb Petie Salpetriere presenta gran afinidad por el oxígeno (incrementada 10 veces), una cooperatividad reducida, un efecto Bohr reducido y un efecto normal o levemente reducido del 2,3-difosfoglicerato. En términos generales, las variantes de afinidad elevada se saturan de oxígeno a nivel pulmonar, pero en el capilar tisular a una pO2 entre 35 y 45 mm Hg, liberan menos oxígeno que la Hb A normal, lo que resulta en una hipoxia tisular leve que estimula una mayor producción de eritropoyetina y la consiguiente policitemia [5, 6].

Las hemoglobinopatías con una afinidad por el oxígeno alterada son enfermedades infrecuentes, aunque su sospecha puede surgir en pruebas analíticas sencillas como la determinación de la p50. En nuestra opinión, este parámetro se debería analizar de manera sistemática en pacientes con eritrocitosis, especialmente en pacientes jóvenes en los que la policitemia vera se considera estadísticamente improbable, o al menos después de que se haya descartado mediante criterios clínicos y analíticos 5], [6], [7.

## Lecciones aprendidas

Se trata del primer caso de detección de la variante Hb Petie Salpetriere en un paciente español. Los resultados obtenidos indican que la variante Hb Petie Salpetriere puede interferir en los resultados de los análisis de HbA_1c_ realizados mediante HPLC de intercambio iónico, no ocurriendo lo mismo con el método de inmunoensayo de aglutinación de látex. La inclusión de niveles de glucosa en plasma puede resultar útil a la hora de interpretar los valores de HbA_1c_. Así mismo, únicamente el HPLC de intercambio iónico reveló la presencia de la variante de la Hb en este caso, lo que indica que se puede detectar una posible variante mediante una revisión meticulosa del cromatograma. Finalmente, la presencia de una variante de la Hb puede ser confirmada mediante estudios genéticos.
